# The Role of D-allulose and Erythritol on the Activity of the Gut Sweet Taste Receptor and Gastrointestinal Satiation Hormone Release in Humans: A Randomized, Controlled Trial

**DOI:** 10.1093/jn/nxac026

**Published:** 2022-02-04

**Authors:** Fabienne Teysseire, Valentine Bordier, Aleksandra Budzinska, Nathalie Weltens, Jens F Rehfeld, Jens J Holst, Bolette Hartmann, Christoph Beglinger, Lukas Van Oudenhove, Bettina K Wölnerhanssen, Anne Christin Meyer-Gerspach

**Affiliations:** St. Clara Research Ltd at St. Claraspital, Basel, Switzerland; Faculty of Medicine, University of Basel, Basel, Switzerland; St. Clara Research Ltd at St. Claraspital, Basel, Switzerland; Faculty of Medicine, University of Basel, Basel, Switzerland; Laboratory for Brain-Gut Axis Studies, Translational Research Center for Gastrointestinal Disorders, Department of Chronic Diseases and Metabolism, KU Leuven, Leuven, Belgium; Leuven Brain Institute, KU Leuven, Leuven, Belgium; Laboratory for Brain-Gut Axis Studies, Translational Research Center for Gastrointestinal Disorders, Department of Chronic Diseases and Metabolism, KU Leuven, Leuven, Belgium; Leuven Brain Institute, KU Leuven, Leuven, Belgium; Department of Clinical Biochemistry, Rigshospitalet, University of Copenhagen, Copenhagen, Denmark; Department of Biomedical Sciences and Novo Nordisk Foundation Center for Basic Metabolic Research, Faculty of Health and Medical Sciences, University of Copenhagen, Copenhagen, Denmark; Department of Biomedical Sciences and Novo Nordisk Foundation Center for Basic Metabolic Research, Faculty of Health and Medical Sciences, University of Copenhagen, Copenhagen, Denmark; St. Clara Research Ltd at St. Claraspital, Basel, Switzerland; Laboratory for Brain-Gut Axis Studies, Translational Research Center for Gastrointestinal Disorders, Department of Chronic Diseases and Metabolism, KU Leuven, Leuven, Belgium; Leuven Brain Institute, KU Leuven, Leuven, Belgium; Cognitive and Affective Neuroscience Lab, Department of Psychological and Brain Sciences, Dartmouth College, Hanover, NH, USA; St. Clara Research Ltd at St. Claraspital, Basel, Switzerland; Faculty of Medicine, University of Basel, Basel, Switzerland; St. Clara Research Ltd at St. Claraspital, Basel, Switzerland; Faculty of Medicine, University of Basel, Basel, Switzerland

**Keywords:** D-allulose, erythritol, gut sweet taste receptor, lactisole, gastrointestinal satiation hormones, gastric emptying, appetite-related sensations

## Abstract

**Background:**

Glucose induces the release of gastrointestinal (GI) satiation hormones, such as glucagon-like peptide 1 (GLP-1) and peptide tyrosine tyrosine (PYY), in part via the activation of the gut sweet taste receptor (T1R2/T1R3).

**Objectives:**

The primary objective was to investigate the importance of T1R2/T1R3 for the release of cholecystokinin (CCK), GLP-1, and PYY in response to D-allulose and erythritol by assessing the effect of the T1R2/T1R3 antagonist lactisole on these responses and as secondary objectives to study the effect of the T1R2/T1R3 blockade on gastric emptying, appetite-related sensations, and GI symptoms.

**Methods:**

In this randomized, controlled, double-blind, crossover study, 18 participants (5 men) with a mean ± SD BMI (in kg/m^2^) of 21.9 ± 1.7 and aged 24 ± 4 y received an intragastric administration of 25 g D-allulose, 50 g erythritol, or tap water, with or without 450 parts per million (ppm) lactisole, respectively, in 6 different sessions. ^13^C-sodium acetate was added to all solutions to determine gastric emptying. At fixed time intervals, blood and breath samples were collected, and appetite-related sensations and GI symptoms were assessed. Data were analyzed with linear mixed-model analysis.

**Results:**

D-allulose and erythritol induced a significant release of CCK, GLP-1, and PYY compared with tap water (all *P*_Holm_ < 0.0001, *d_z_* >1). Lactisole did not affect the D-allulose– and erythritol-induced release of CCK, GLP-1, and PYY (all *P*_Holm_ > 0.1). Erythritol significantly delayed gastric emptying, increased fullness, and decreased prospective food consumption compared with tap water (*P*_Holm_ = 0.0002, *d_z_* = –1.05; *P*_Holm_ = 0.0190, *d_z_* = 0.69; and *P*_Holm_ = 0.0442, *d_z_* = –0.62, respectively).

**Conclusions:**

D-allulose and erythritol stimulate the secretion of GI satiation hormones in humans. Lactisole had no effect on CCK, GLP-1, and PYY release, indicating that D-allulose– and erythritol-induced GI satiation hormone release is not mediated via T1R2/T1R3 in the gut.

## Introduction

The increasing prevalence of obesity and diabetes mellitus type 2 (T2DM) and associated metabolic and cardiovascular disorders creates serious health problems worldwide (1). Sugar consumption has been shown to have harmful effects on the development of these diseases (2, 3). The WHO strongly recommends to reduce free sugar intake to <10% of total energy intake, preferably <5% (4). Partial substitution of sugar with natural, low-caloric sweeteners such as D-allulose and erythritol is one possible way to achieve the WHO recommendations.

Enteroendocrine cells (EECs) form the largest endocrine organ in the body, although they represent only 1% of the epithelial cells in the gut (5). Scattered along the gastrointestinal (GI) tract, they are responsible for nutrient sensing, resulting in the release of GI satiation hormones such as cholecystokinin (CCK), glucagon-like peptide 1 (GLP-1), and peptide tyrosine tyrosine (PYY) (6). These hormones signal retardation of gastric emptying, increases in satiety and fullness, and reduction in food intake (7–12). In humans, glucose can induce the release of GI satiation hormones via the activation of the sweet taste receptor (T1R2/T1R3) located on EECs (13), whereas this is not the case for artificial sweeteners, such as sucralose, acesulfame K, or cyclamate (14–16). Lactisole, a competitive inhibitor of the T1R3 subunit, attenuates glucose-stimulated release of GLP-1 and PYY in humans (13, 17).

D-allulose (C-3 epimer of D-fructose), also known as D-psicose, is a natural sugar with zero calories (18) and 70% of the sweetness of sucrose. In nature, it occurs only in small amounts, but it is industrially produced by enzymes catalyzing the conversion of D-fructose into D-allulose (19). Moreover, D-allulose seems to have beneficial effects regarding fat and glucose metabolism in humans (20–23). Animal studies have indicated GLP-1 release upon D-allulose administration (24, 25). The effect of D-allulose on GI satiation hormone release and on gastric emptying is not yet known in humans.

Erythritol is a naturally occurring sugar-alcohol without calories and 70% of the sweetness of sucrose, which can be commercially produced by yeast fermentation of glucose. Besides the preventive effect on caries (26), erythritol has a glycemic index of zero (27). Recently, we demonstrated that intragastric administration of erythritol induced the release of CCK, GLP-1, and PYY similar to glucose in healthy participants. Furthermore, erythritol leads to a significant retardation of gastric emptying (28, 29). Whether D-allulose induces the release of GI satiation hormones and, if yes, whether their secretion is mediated via T1R2/T1R3 has not been studied in humans. Also, whether the erythritol-induced GI satiation hormone secretion is mediated via the gut sweet taste receptor is not yet known.

The primary objective of this study was therefore to investigate the importance of T1R2/T1R3 for the release of CCK, GLP-1, and PYY in response to intragastric administration of D-allulose and erythritol in healthy humans by assessing the effect of lactisole on these responses. The secondary objectives aimed to study the effect of the T1R2/T1R3 blockade on gastric emptying, appetite-related sensations, and GI symptoms. More specifically, we hypothesize that CCK, GLP-1, and PYY will be released in response to D-allulose and erythritol compared with tap water. We also hypothesize that GLP-1 and PYY but not CCK release will be reduced by lactisole. Gastric emptying rates will be reduced in response to D-allulose and erythritol compared with tap water, without an effect of lactisole. Satiety/fullness and hunger/prospective food consumption will be increased and reduced, respectively, in response to D-allulose and erythritol compared with tap water, without an effect of lactisole.

## Methods

### Participants

**FIGURE 1:**
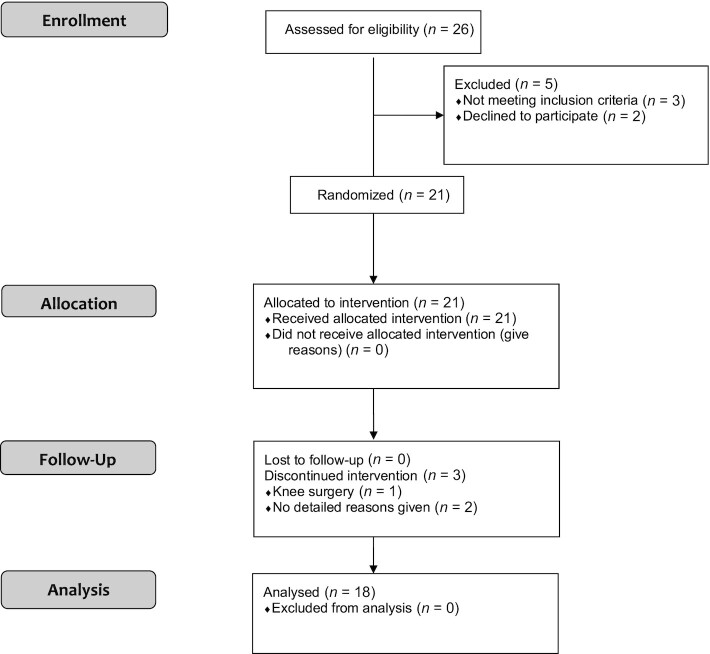
CONSORT flow diagram.

A total of 18 normal-weight, healthy participants (5 men and 13 women) with a mean ± SD BMI (in kg/m^2^) of 21.9 ± 1.7 (range: 19.1–24.3) and aged 24 ± 4 y (range: 19–39 y) completed the study. See participant flowchart in **Figure 1**.

### Overall study design

The study was conducted as a randomized (counterbalanced), placebo-controlled, double-blind, crossover trial. The protocol was approved by the Ethics Committee of Basel, Switzerland (Ethikkomission Nordwest- und Zentralschweiz: 2019–01,111) and conducted in accordance with the principles of the Declaration of Helsinki (version October 2013), the International Conference on Harmonisation for Good Clinical Practice (ICH-GCP), and national legal and regulatory requirements. Recruitment of participants and follow-up took place over a period of 12 mo (September 2019 to September 2020). Each participant gave written informed consent for the study. The study was registered at clinicaltrials.gov as NCT04027283. Exclusion criteria included substance and alcohol abuse, acute infections, chronic medical illness, or illnesses affecting the GI system. None of the participants had a history of food allergies, dietary restrictions, or preexisting consumption of D-allulose and/or erythritol more than once a week. Weight, height, BMI, heart rate, and blood pressure were recorded for all participants. On 6 separate test sessions, at least 3 d apart and after a 10-h overnight fast, participants were admitted to the St. Clara Research Ltd at ∼08:30 h. An antecubital catheter was inserted into a forearm vein for blood collection. Participants swallowed a polyvinyl feeding tube (external diameter 8 French). The tube was introduced via an anesthetized nostril. The rationale for intragastric administration of the test solutions was to bypass orosensory cues to provide information on the isolated postoral effects, which is crucial to increase the understanding of the role of the GI tract in the short-term control of appetite without confounding effects of cephalic and oral phases of ingestion, triggering hedonic responses and cognitions.

### Experimental procedure

After taking blood samples (t = –10 and –1 min) and breath samples (t = –10 min) in the fasting state, as well as recording of appetite-related sensations and GI symptoms, participants received one of the following test solutions (at t = 0 min) directly into the stomach over 2 min in a randomized order:

- 50 g erythritol dissolved in 300 mL tap water
- 50 g erythritol and 450 parts per million (ppm) lactisole dissolved in 300 mL tap water
- 25 g D-allulose dissolved in 300 mL tap water
- 25 g D-allulose and 450 ppm lactisole dissolved in 300 mL tap water
- 300 mL tap water (placebo)
- 300 mL tap water and 450 ppm lactisole (placebo)

Concentrations were chosen based on the following considerations: 50 g erythritol induces GI satiation hormone release reliably without GI side effects and corresponds to ∼33.5 g sucrose typically found in sweet beverages (28). The effect of D-allulose on GI satiation hormones has not been investigated so far. The recommended maximal single dose—where no GI side effects are observed—is 25 g (30). In a previous study design, 450 ppm lactisole reliably induces a blockade of the gut sweet taste receptor (13). The effectiveness of lactisole has been tested before in a pretest oral taste experiment. Lactisole was able to block the D-allulose– and erythritol-induced sweet taste on the tongue. The results are in line with previous observations of other sweeteners (31). To determine gastric emptying rates, 50 mg ^13^C-sodium acetate was added to the different test solutions. The intragastric test solutions were freshly prepared each morning of the study and were at room temperature when administered. The participants and the personnel involved in performing the study days and blood analysis were blinded regarding the content of administered test solutions.

After the administration of the test solution, blood samples (at t = 15, 30, 45, 60, 90, 120, and 180 min), for analysis of plasma CCK, GLP-1, and PYY, and end-expiratory breath samples (at t = 15, 30, 45, 60, 75, 90, 105, 120, 150, 180, 210, and 240 min), for analysis of gastric emptying rates, were taken.

Appetite-related sensations (hunger, prospective food consumption, satiety, and fullness) were assessed at t = 15, 30, 45, 60, 90, 120, and 180 min using visual analog scales (VASs) as previously described (32, 33). The ratings were recorded to 1 decimal point (e.g., 2.1).

Participants were also asked to rate GI symptoms [no symptoms (0 points), mild (1 point), or severe symptoms (2 points)] at t = 30, 60, 90, 120, 150, 180, and 240 min after the administration of the test solutions. The list included the following symptoms: abdominal pain, nausea, vomiting, diarrhea, borborygmus, abdominal bloating, eructation, and flatulence.

Vital signs (blood pressure, heart rate) were measured at the beginning and at the end of each study day.

### Materials

Erythritol was purchased from Mithana GmbH and ^13^C-sodium acetate from ReseaChem. D-allulose was purchased from Tate&Lyle. Lactisole was a friendly gift of Domino Sugar Corporation.

### Blood sample collection and processing

CCK, GLP-1, and PYY blood samples were collected on ice into tubes containing EDTA (6 μmol/L blood), a protease inhibitor cocktail (Complete, EDTA free, 1 tablet/50 mL blood; Roche), and a dipeptidyl peptidase IV inhibitor (10 μL/mL blood; Millipore). After centrifugation (4°C at 1409 × *g*for 10 min), plasma samples were immediately processed into different aliquots and stored at –80°C until analysis.

### Assessment of gastric emptying

The gastric emptying rate was determined using a ^13^C-sodium acetate test, an accurate, noninvasive method for measuring gastric emptying, without radiation exposure, and a reliable alternative to scintigraphy, the current “gold standard” (34). Test solutions were enriched with 50 mg ^13^C-sodium acetate, a compound readily absorbed in the proximal small intestine and transported to the liver, where it is metabolized to ^13^CO_2_, which is then exhaled rapidly (34). At t = –10, 15, 30, 45, 60, 75, 90, 105, 120, 150, 180, 210, and 240 min, end-expiratory breath samples were taken into a 100-mL foil bag. The ^13^C-exhalation was determined by nondispersive infrared spectroscopy using an isotope ratio mass spectrophotometer (Kibion Dynamic Pro; Kibion GmbH) and expressed as the relative difference (δ ‰) from the universal reference standard (carbon from Pee Dee Belemnite limestone). ^13^C-enrichment was defined as the difference between preprandial ^13^C-exhalation and postprandial ^13^C-exhalation at defined time points, δ over basal (‰). Delta values were converted into atom percent excess and then into percentage of administered dose of ^13^C excreted per hour [% dose/h (%)].

### Laboratory analysis

Plasma CCK was measured with a sensitive radioimmunoassay using a highly specific antiserum (No. 92,128) (35). The intra- and interassay variability is <15%, respectively. The appropriate range of this assay is 0.1 to 20 pmol/L. Plasma GLP-1 samples were extracted in a final concentration of 70% ethanol before GLP-1 analysis. Total GLP-1 was measured as described by Ørskov et al. (36) using a radioimmunoassay (antibody code No. 89,390) specific for the C-terminal part of the GLP-1 molecule and reacting equally with intact GLP-1 and the primary (N-terminally truncated) metabolite. The intra-assay variability is <10%, and the sensitivity of this assay is <1 pmol/L. Plasma PYY was measured using Millipore human total PYY ELISA (cat. EZHPYYT66K; Millipore). The intra- and interassay variability is <5.78% and 16.5%, respectively. The dynamic range of this assay is 14 pg/mL to 1800 pg/mL when using a 20-μL sample size.

### Statistical analysis

In previous data on GI satiation hormone responses to intragastric infusion of 50 g erythritol compared with tap water (29), the smallest proposed sample size (*N* = 18) yields 100% power to detect the hypothesized difference in the CCK, GLP-1, and PYY response between erythritol and tap water in linear mixed-model analyses. Based on previous data on lactisole inhibition of glucose-induced hormone secretion (13), *N* = 18 yields >80% power to detect the hypothesized inhibitory effect of lactisole on GLP-1 and PYY secretion. Data were analyzed in SAS 9.4 (SAS Institute) and shown as mean ± SEM unless otherwise stated. A 2-tailed *P* value ≤ 0.05 was considered significant and Cohen's *d_z_* for paired *t* tests was reported as a measure of effect size.

For all analyses, if the assumption of normally distributed residuals was violated (based on a significant *P* value of the Shapiro–Wilk test), natural logarithmic transformations of the dependent variables were used to normalize this distribution. Analysis was performed on transformed data. Logarithmic transformation of the dependent variables adequately normalized the residual distribution. Visit number was included to control for putative order effects in all models. All outcome variables were analyzed using (generalized) linear mixed models on changes from baseline [average of preinfusion time point(s)]. “Test solution” (intragastric D-allulose, D-allulose + lactisole, erythritol, erythritol + lactisole, tap water, and tap water + lactisole) and “time” were included as within-subject independent variables in the models (including their main effects and the interaction). All the models were controlled for baseline values. To follow up on significant main or interaction effects, planned contrast analyses were performed to test our specific hypotheses, with stepdown Bonferroni (Holm) correction for multiple testing. To test the hypothesis that D-allulose or erythritol induces an increase in GI satiation hormones and retards gastric emptying compared with tap water, we compared postinfusion GI satiation hormone concentrations and gastric emptying (change from baseline) between tap water, on one hand, and D-allulose or erythritol, on the other hand. To test the hypothesis that D-allulose or erythritol increases satiety/fullness and decreases hunger/prospective food consumption compared with tap water, respectively, we compared postinfusion appetite-related sensations between tap water, on one hand, and D-allulose or erythritol, on the other hand. To test the hypothesis that addition of lactisole does (not) decrease GI satiation hormones, retard gastric emptying, or change appetite-related sensations in response to D-allulose or erythritol, we compared postinfusion GI satiation hormone concentrations and gastric emptying (change from baseline) to each of the substances with and without added lactisole.

For the associations, the difference between the test solutions of the significant planned contrasts at each time point was calculated and used as a dependent variable in the model with the same difference at each time point for the GI satiation hormones as an independent variable in addition to time.

## Results

Twenty-one participants were recruited for the study. There were 3 dropouts (1 participant had to withdraw due to a knee surgery and 2 withdrew for personal reasons). Therefore, 18 participants completed the 6 treatments. Complete data from all 18 participants were available for analysis.

### GI satiation hormones

#### Plasma CCK, GLP-1, and PYY

**FIGURE 2:**
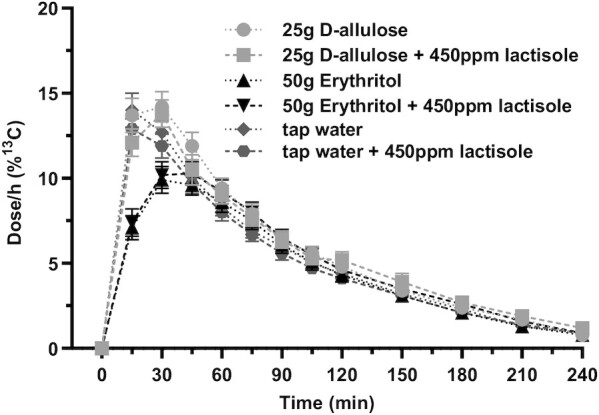
(A) CCK, (B) GLP-1, and (C) PYY release after intragastric administration of solutions containing 25 g D-allulose, 25 g D-allulose + 450 ppm lactisole, 50 g erythritol, 50 g erythritol + 450 ppm lactisole, tap water, and tap water + 450 ppm lactisole to 18 healthy adults. Data are expressed as mean ± SEM; absolute values are reported. *N* = 18 (5 men, 13 women). Statistical tests: linear mixed-effects modeling followed by planned contrasts with Holm correction for multiple testing. The increase of CCK, GLP-1, and PYY was greater for D-allulose and erythritol compared with tap water (comparisons of the changes from baseline, all *P*_Holm_ < 0.0001, *d_z_* >1), with no significant difference for D-allulose + lactisole and erythritol + lactisole compared with the test solutions without lactisole (all *P*_Holm_ > 0.1). CCK, cholecystokinin; GLP-1, glucagon-like peptide-1; ppm, parts per million; PYY, peptide tyrosine tyrosine.

CCK, GLP-1, and PYY secretion in response to D-allulose and erythritol is depicted in **Figure 2** and **Table 1**. Both D-allulose and erythritol induced a significant increase in GI satiation hormones compared with tap water. Adding lactisole had no effect on the secretion.

**TABLE 1:** Estimates from linear mixed models, results from planned contrast analyses, and effect sizes in response to intragastric administration of solutions containing 25 g D-allulose, 25 g D-allulose + 450 ppm lactisole, 50 g erythritol, 50 g erythritol + 450 ppm lactisole, tap water, and tap water + 450 ppm lactisole to 18 healthy adults^1^

	Test solutions				*P* values	
Characteristic	D-allulose vs. tap water	D-allulose vs. D-allulose + lactisole	Erythritol vs. tap water	Erythritol vs. erythritol + lactisole	Main effect of test solution	Test solution-by-time interaction
CCK, pmol/L	0.77 ± 0.12	–0.29 ± 0.16	1.58 ± 0.19	0.03 ± 0.23	<0.0001	<0.0001
* P*_Holm_	<0.0001	0.1703	<0.0001	0.8800		
* d_z_*	1.48		1.94			
GLP-1, pmol/L	4.08 ± 0.76	0.55 ± 1.09	7.41 ± 0.96	2.40 ± 1.34	<0.0001	0.0203
* P*_Holm_	<0.0001	0.6136	<0.0001	0.1594		
* d_z_*	1.27		1.83			
PYY, pg/mL	64.4 ± 6.15	9.48 ± 6.35	104 ± 9.21	13.5 ± 8.68	<0.0001	<0.0001
* P*_Holm_	<0.0001	0.2502	<0.0001	0.2502		
* d_z_*	2.47		2.67			
Gastric emptying, dose/h(%^13^C)	0.10 ± 0.16	–0.22 ± 0.28	–0.37 ± 0.08	0.08 ± 0.17	0.0003	<0.0001
* P*_Holm_	1	1	0.0002	1		
* d_z_*			–1.05			
Hunger, cm	–0.14 ± 0.24	0.56 ± 0.31	–0.49 ± 0.25	0.16 ± 0.28	0.2020	0.0400
* P*_Holm_	0.2283	1	0.2283	1		
Pfc, cm	0.06 ± 0.21	0.39 ± 0.32	–0.61 ± 0.24	–0.08 ± 0.27	0.0615	0.1784
* P*_Holm_	0.6811	1	0.0442	1		
* d_z_*			–0.62			
Satiety, cm	–0.23 ± 0.26	0.18 ± 0.27	0.47 ± 0.29	0.41 ± 0.36	0.1206	0.1521
* P*_Holm_	0.7695	0.7695	0.4533	0.7695		
Fullness, cm	–0.18 ± 0.22	0.16 ± 0.33	0.71 ± 0.24	0.62 ± 0.33	0.0011	0.3473
* P*_Holm_	0.8714	0.8714	0.0190	0.2071		
* d_z_*			0.69			

1 *N* = 18 (5 men, 13 women). Estimates are expressed as mean ± SE and represent the changes from baseline for D-allulose and erythritol compared with tap water and the changes from baseline for lactisole within D-allulose and erythritol. Statistical tests: linear mixed-effects modeling followed by planned contrasts with Holm correction for multiple testing and Cohen's *d_z_* for paired *t* tests is reported as a measure of effect size. CCK, cholecystokinin; GLP-1, glucagon-like peptide 1; Pfc, prospective food consumption; ppm, parts per million; PYY, peptide tyrosine tyrosine.

Planned contrast analyses showed that the increase of CCK, GLP-1, and PYY was greater for D-allulose and erythritol compared with tap water (comparisons of the changes from baseline, all *P*_Holm_ < 0.0001, *d_z_* >1), with no significant difference for D-allulose + lactisole and erythritol + lactisole compared with the test solutions without lactisole (all *P*_Holm_ > 0.1). The main effect of test solution was significant for CCK, GLP-1, and PYY [*F*(5, 65) = 14.08, *P* < 0.0001; *F*(5, 60) = 12.85, *P* < 0.0001; and *F*(5, 54) = 28.68, *P* < 0.0001, respectively], indicating a difference in GI satiation hormone concentrations between the 6 test solutions over all time points. Furthermore, the test solution-by-time interaction effect was significant for CCK, GLP-1, and PYY [*F*(30, 264) = 7.73, *P* < 0.0001; *F*(30, 267) = 1.66, *P* = 0.0203; and *F*(30, 271) = 5.26, *P* < 0.0001, respectively], indicating that the difference between test solutions differs between time points.

### Gastric emptying

**FIGURE 3:**
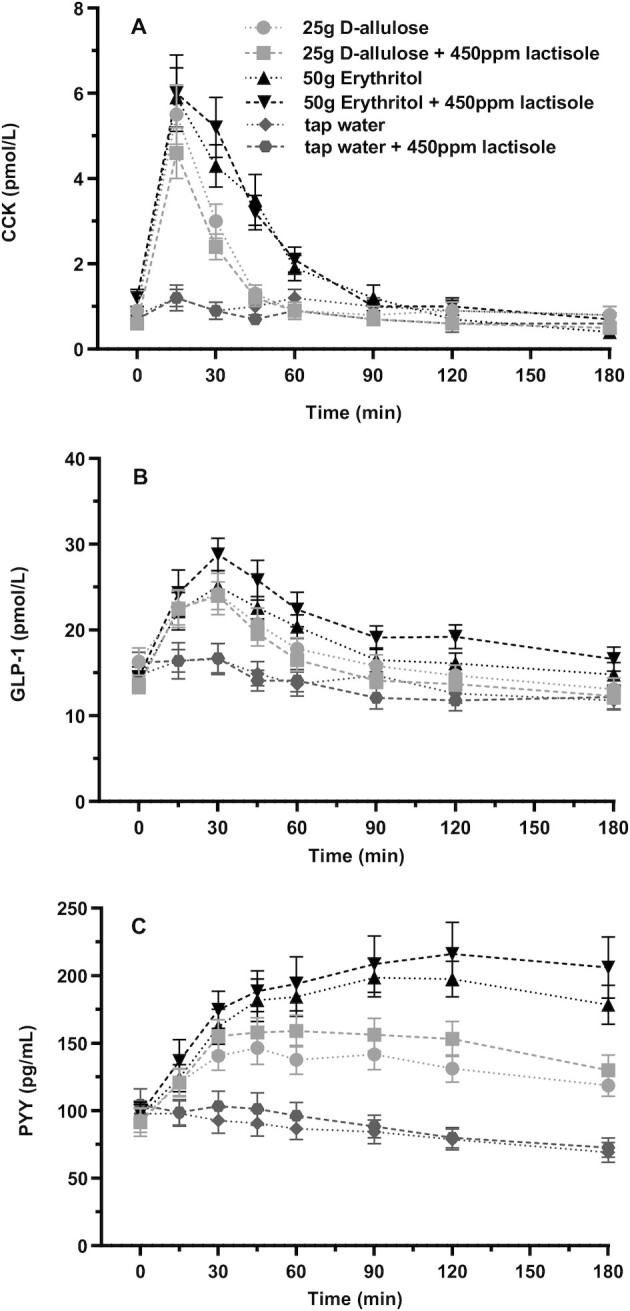
Gastric emptying after intragastric administration of solutions containing 25 g D-allulose, 25 g D-allulose + 450 ppm lactisole, 50 g erythritol, 50 g erythritol + 450 ppm lactisole, tap water, and tap water + 450 ppm lactisole to 18 healthy adults. Data are expressed as mean ± SEM. Change from baseline values is reported. *N* = 18 (5 men and 13 women). Statistical tests: linear mixed-effects modeling followed by planned contrasts with Holm correction for multiple testing. Gastric emptying was retarded for erythritol compared with tap water but not for D-allulose compared with tap water (comparisons of the changes from baseline, *P*_Holm_ = 0.0002, *d_z_* = –1.05 and *P*_Holm_ = 1, respectively), with no significant difference for D-allulose + lactisole and erythritol + lactisole compared with the test solutions without lactisole (all *P*_Holm_ = 1). CCK, cholecystokinin; GLP-1, glucagon-like peptide-1; ppm, parts per million; PYY, peptide tyrosine tyrosine.

Changes in gastric emptying in response to D-allulose and erythritol are depicted in **Figure 3** and Table 1. Erythritol induced a significant retardation of gastric emptying compared with tap water, whereas D-allulose had no effect. Adding lactisole did not retard gastric emptying. Planned contrast analyses showed that gastric emptying was retarded for erythritol compared with tap water but not for D-allulose compared with tap water (comparisons of the changes from baseline, *P*_Holm_ = 0.0002, *d_z_* = –1.05 and *P*_Holm_ = 1, respectively), with no significant difference for D-allulose + lactisole and erythritol + lactisole compared with the test solutions without lactisole (all *P*_Holm_ = 1). The main effect of test solution was significant [*F*(5, 39) = 6.13, *P *= 0.0003], indicating a difference in gastric emptying between the 6 test solutions over all time points. Furthermore, the test solution-by-time interaction effect was significant [*F*(15, 102) = 10.43, *P* < 0.0001], indicating that the difference between test solutions differs between time points.

### Appetite-related sensations

#### Hunger

**FIGURE 4:**
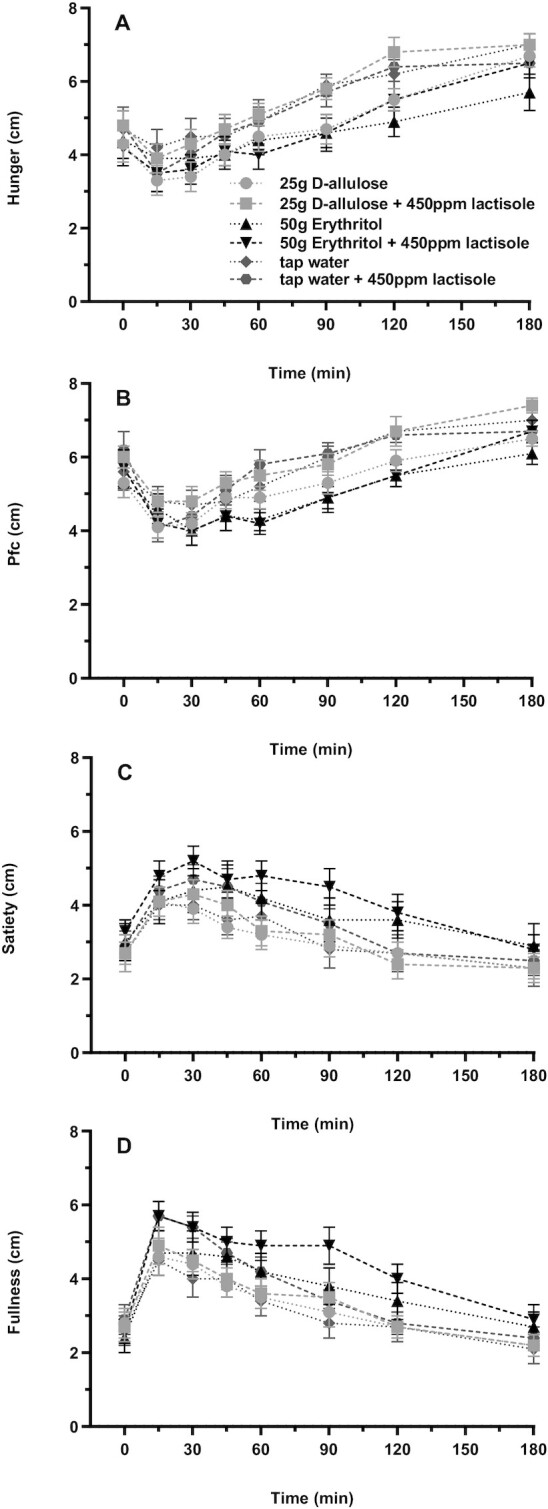
(A) Hunger, (B) Pfc, (C) satiety, and (D) fullness after intragastric administration of solutions containing 25 g D-allulose, 25 g D-allulose + 450 ppm lactisole, 50 g erythritol, 50 g erythritol + 450 ppm lactisole, tap water, and tap water + 450 ppm lactisole to 18 healthy adults. Data are expressed as mean ± SEM; absolute values are reported. *N* = 18 (5 men and 13 women). Statistical tests: linear mixed-effects modeling followed by planned contrasts with Holm correction for multiple testing. Pfc was lower for erythritol compared with tap water but not for D-allulose compared with tap water (comparisons of the changes from baseline, *P*_Holm_ = 0.0442, *d_z_* = –0.60 and *P*_Holm_ = 0.6811, respectively), with no significant difference for D-allulose + lactisole and erythritol + lactisole compared with the test solutions without lactisole (both *P*_Holm_ = 1). Fullness was greater for erythritol compared with tap water but not for D-allulose compared with tap water (comparisons of the changes from baseline, *P*_Holm_ = 0.0190, *d_z_* = 0.69 and *P*_Holm_ = 0.8714, respectively), with no significant difference for D-allulose + lactisole, and erythritol + lactisole compared with the test solutions without lactisole (*P*_Holm_ = 0.9814 and *P*_Holm_ = 0.2071, respectively). No significant results for hunger and satiety. Pfc, prospective food consumption; ppm, parts per million.

Sensations of hunger in response to D-allulose and erythritol are depicted in **Figure 4**A and Table 1. Neither D-allulose nor erythritol affected the sensations of hunger compared with tap water. Adding lactisole had no effect. None of the planned contrast analyses were significant. The main effect of test solution was not significant [*F*(5, 57) = 1.51, *P* = 0.2020], indicating no difference in hunger between the 6 test solutions over all time points. Furthermore, the test solution-by-time interaction effect was significant [*F*(30, 277) = 1.54, *P* = 0.0400].

#### Prospective food consumption

Sensations of prospective food consumption in response to D-allulose and erythritol are depicted in Figure 4B and Table 1. Erythritol decreased the sensations of prospective food consumption compared with tap water, whereas D-allulose had no effect. Adding lactisole had no effect. Planned contrast analyses showed that prospective food consumption was lower for erythritol compared with tap water but not for D-allulose compared with tap water (comparisons of the changes from baseline, *P*_Holm_ = 0.0442, *d_z_* = –0.62 and *P*_Holm_ = 0.6811, respectively), with no significant difference for D-allulose + lactisole and erythritol + lactisole compared with the test solutions without lactisole (both *P*_Holm_ = 1). Neither the main effect of test solution [*F*(5, 61) = 2.24, *P* = 0.0615] nor the test solution-by-time interaction effect [*F*(30, 278) = 1.25, *P* = 0.1784] was significant.

#### Satiety

Sensations of satiety in response to D-allulose and erythritol are depicted in Figure 4C and Table 1. Neither D-allulose nor erythritol affected the sensations of satiety compared with tap water. Adding lactisole had no effect. None of the planned contrast analyses were significant.

Neither the main effect of test solution [*F*(5, 51) = 1.84, *P* = 0.1206] nor the test solution-by-time interaction effect [*F*(30, 283) = 1.29, *P* = 0.1521] was significant.

#### Fullness

Sensations of fullness in response to D-allulose and erythritol are depicted in Figure 4D and Table 1. Erythritol increased the sensations of fullness compared with tap water, whereas D-allulose had no effect. Adding lactisole had no effect. Planned contrast analyses showed that fullness was greater for erythritol compared with tap water but not for D-allulose compared with tap water (comparisons of the changes from baseline, *P*_Holm_ = 0.0190, *d_z_* = 0.69 and *P*_Holm_ = 0.8714, respectively), with no significant difference for D-allulose + lactisole and erythritol + lactisole compared with the test solutions without lactisole (*P*_Holm_ = 0.9814 and *P*_Holm_ = 0.2071, respectively). The main effect of test solution was significant [*F*(5, 55) = 4.76, *P* = 0.0011], indicating a difference in fullness between the 6 test solutions over all time points. Furthermore, the test solution-by-time interaction effect was not significant [*F*(30, 280) = 1.09, *P* = 0.3473].

### Associations between GI satiation hormones and gastric emptying

The difference in GLP-1 concentrations between erythritol and tap water was significantly associated with the respective difference in gastric emptying [β ± SE: 0.05 ± 0.02; *F*(1, 101) = 7.33, *P* = 0.0080 *d_z_* = 0.64]. The differences in CCK and PYY concentrations between erythritol and tap water were not associated with the respective difference in gastric emptying [0.04 ± 0.06, *F*(1, 101) = 0.4, *P* = 0.5301 and 0.001 ± 0.003, *F*(1, 101) = 0.59, *P* = 0.4449, respectively].

### Associations between GI satiation hormones and appetite-related sensations

The difference in GLP-1 concentrations between erythritol and tap water was significantly associated with the respective difference in prospective food consumption [–0.06 ± 0.02, *F*(1, 101) = 5.60, *P* = 0.0199, *d_z_* = –0.64]. The differences in CCK and PYY concentrations between erythritol and tap water were not associated with the respective difference in prospective food consumption [–0.06 ± 0.07, *F*(1, 101) = 0.88, *P* = 0.3501 and 0.00002 ± 0.004, *F*(1, 101) = 0.00, *P* = 0.9956, respectively]. The differences in CCK, GLP-1, and PYY concentrations between erythritol and tap water were not associated with the respective difference in fullness [0.07 ± 0.06, *F*(1, 101) = 1.08, *P* = 0.3009; 0.008 ± 0.02, *F*(1, 101) = 0.11, *P* = 0.7458; and 0.003 ± 0.004, *F*(1, 101) = 0.85, *P* = 0.3597, respectively].

### Gastrointestinal symptoms

All participants tolerated the study well. None of the participants had to withdraw from the study due to GI-related symptoms. The symptoms were mild and short-lasting. Details are listed in **Table 2**.

**TABLE 2:** Assessment of gastrointestinal symptoms after intragastric administration of solutions containing 25 g D-allulose, 25 g D-allulose + 450 ppm lactisole, 50 g erythritol, 50 g erythritol + 450 ppm lactisole, tap water, and tap water + 450 ppm lactisole to 18 healthy adults^1^

Symptom	Participants with symptom, *n*^2^	Reported severity^3^
Abdominal pain		
D-allulose	3	1.0
D-allulose + lactisole	4	1.0
Erythritol	6	1.0
Erythritol + lactisole	7	1.0
Tap water	2	1.0
Tap water + lactisole	3	1.0
Nausea		
D-allulose	3	1.0
D-allulose + lactisole	3	1.0
Erythritol	9	1.0
Erythritol + lactisole	10	1.1
Tap water	1	1.0
Tap water + lactisole	3	1.3
Vomiting		
D-allulose	0	0
D-allulose + lactisole	0	0
Erythritol	2	1.5
Erythritol + lactisole	0	0
Tap water	0	0
Tap water + lactisole	0	0
Diarrhea		
D-allulose	2	1.0
D-allulose + lactisole	0	0.0
Erythritol	5	1.0
Erythritol + lactisole	3	1.3
Tap water	0	0
Tap water + lactisole	0	0
Bowel sounds		
D-allulose	11	1.1
D-allulose + lactisole	13	1.1
Erythritol	14	1.0
Erythritol + lactisole	14	1.1
Tap water	11	1.0
Tap water + lactisole	8	1.0
Bloating		
D-allulose	3	1.0
D-allulose + lactisole	3	1.0
Erythritol	5	1.0
Erythritol + lactisole	4	1.0
Tap water	2	1.0
Tap water + lactisole	0	1.0
Eructation		
D-allulose	4	1.0
D-allulose + lactisole	4	1.0
Erythritol	4	1.0
Erythritol + lactisole	7	1.3
Tap water	2	1.0
Tap water + lactisole	3	1.0
Flatulence		
D-allulose	3	1.0
D-allulose + lactisole	2	1.0
Erythritol	5	1.0
Erythritol + lactisole	3	1.0
Tap water	0	0
Tap water + lactisole	0	0

1 *N* = 18 (5 men, 13 women). ppm, parts per million.

2 Gastrointestinal symptoms were assessed by the use of a list. Participants were asked to choose between “no symptom” (0 points), “mild symptoms” (1 point), and “severe symptoms” (2 points) for each item.

3 Reported severity was calculated by the sum of the points divided by the participants with symptom.

## Discussion

The results of the current study can be summarized as follows: D-allulose and erythritol induced a statistically significant release of CCK, GLP-1, and PYY compared with tap water. Lactisole did not affect the D-allulose– and erythritol-induced release of CCK, GLP-1, and PYY. Erythritol led to a statistically significant retardation of gastric emptying, an increase in fullness, and a decrease in prospective food consumption compared with tap water. Doses of 25 g of D-allulose and 50 g of erythritol were well tolerated.

The increase in obesity and T2DM is related to sugar consumption, especially in the form of sugar-sweetened beverages (2, 3). WHO and other national health institutions have formulated guidelines encouraging consumers to limit their sugar intake (37, 38). A possible way to achieve such reductions in sugar consumption is substitution of sugar with natural, low-caloric sweeteners such as D-allulose and erythritol. Both D-allulose and erythritol may have beneficial effects on glucose metabolism; in addition, both have been shown to stimulate the release of GI satiation hormones (24, 25, 28, 29). Of particular interest are CCK, GLP-1, and PYY, which induce a retardation of gastric emptying, an increase in satiety and fullness, and a reduction in food intake (7–12).

In humans, glucose can induce the release of CCK, GLP-1, and PYY (13), whereas this is not the case for artificial sweeteners, such as sucralose, acesulfame K, or cyclamate (14–16). Here we have shown that intragastric administration of the naturally occurring, low-caloric sweetener D-allulose induces the release of CCK, GLP-1, and PYY in healthy humans, translating rodent studies to humans (24, 25). The previously demonstrated effect of erythritol on the secretion of CCK, GLP-1, and PYY was confirmed in the present study: intragastric administration of 75 g erythritol solution stimulated the secretion of CCK and GLP-1 in healthy participants (28). The findings are in line with the results of Overduin et al. (39), in which the partial replacement of sucrose by erythritol in a test breakfast lead to equal secretion of GLP-1 and PYY.

In humans, glucose has been reported to induce release of GI satiation hormones in part via the activation of T1R2/T1R3; lactisole, a competitive inhibitor of the T1R3 subunit, attenuated the glucose-stimulated release of GLP-1 and PYY, whereas CCK release was unaffected (13). The inhibitory effect of lactisole is specific to humans and other primates (17). We therefore hypothesized that GLP-1 and PYY but not CCK release would be reduced by lactisole in response to D-allulose and erythritol. However, lactisole had no effect on the D-allulose– and erythritol-induced GI satiation hormone release in the current study. The knowledge about the T1R3 blockade in this study is based on the observations made by Schiffman et al. (31) for the sweet taste receptor on the tongue. The sweet intensity of different sweeteners (including sucrose and glucose) was significantly blocked at concentrations of 250 and 500 ppm lactisole. The inhibition was observed only when sweeteners and lactisole were mixed prior to tasting and not when lactisole was introduced prior to these respective substances (31). Therefore, a lack of effect based on mixing the sweeteners with lactisole prior to the intragastric administration can be excluded. Moreover, the use of 450 ppm lactisole is based on previous intragastric studies in which glucose-stimulated secretion of GLP-1 and PYY was significantly reduced (13, 40). In both studies, glucose and lactisole were mixed prior to the intragastric administration. Apart from these studies with lactisole, Karimian Azari et al. (41) used a comparable study design to evaluate the metabolic effects with lactisole in response to an oral glucose load in healthy lean participants with a comparable outcome. Another potential factor that could have interfered with the effectiveness of lactisole inhibition is the relative absorption rates of the test solutions used. D-allulose and erythritol are absorbed with ∼80% and 90% efficiency, respectively, whereas lactisole is rapidly absorbed (42–44). Based on this, lactisole could have effectively blocked the natural sweeteners at the proximal intestine but not at the distal intestine, which may have contributed to the lack of inhibition. However, the distribution and density of GLP-1 cells, although largely distributed in the terminal ileum, is also present in the duodenum (45). Therefore, lactisole should have effectively blocked the sweeteners at the proximal GLP-1 secreting cells, which was not the case.

The lack of effect of lactisole suggests that D-allulose and erythritol induce the release of GI satiation hormones via other receptor/transporter mechanisms. There is evidence suggesting that sodium-dependent glucose cotransporter 1 (SGLT-1) is the main driver of glucose-induced GLP-1 secretion (46). The pharmacologic SGLT-1 inhibitor phlorizin or the comparison between wild-type and *Sglt1^–^^/^^–^* mice reduced glucose-induced GLP-1 release (47–49). However, mice lacking SGLT-1 have an increase in the later phase of GLP-1 secretion after glucose administration alone, suggesting that in the absence of SGLT-1, other pathways are active (50). One hypothesis is that the increased delivery of glucose into the distal intestine possibly involves its fermentation into short-chain fatty acids, which in turn may trigger GLP-1 release (50). Although up to 20% of erythritol is unabsorbed and available for colonic fermentation (42), it is unlikely that this might be a reason for the erythritol-induced GLP-1 release because we have an increase in GLP-1 after 30 min in this study. Furthermore, phlorizin did not reduce D-allulose–induced GLP-1 release in rats, which also contradicts the hypothesis that SGLT-1 plays a role in the GI satiation hormone release (25). In the same study, the authors also used xanthohumol—an inhibitor of the glucose/fructose transporter 5 (GLUT5)—which inhibited D-allulose–induced GLP-1 secretion, suggesting that the secretion might be stimulated via GLUT5. The authors explain this by the fact that D-allulose and fructose are epimers and that a possible mechanism for GLP-1 secretion via GLUT5 has been suggested for fructose (46, 51). Data in humans are lacking so far.

Gastric emptying is regulated by several feedback mechanisms, including GI satiation hormone release such as CCK, GLP-1, and PYY (7, 52). Here erythritol retarded gastric emptying, confirming our previous findings (28, 29). Lactisole had no effect on the erythritol-induced retardation of gastric emptying. The latter findings extend our previous results: Gerspach et al. (13) showed that the retardation of gastric emptying was not affected by lactisole after glucose or after mixed liquid meal administration. We had anticipated that D-allulose would retard gastric emptying—especially in view of the observed effect on the GI satiation hormones—but we were unable to confirm our hypothesis.

Both increased concentrations of GI satiation hormones and prolonged gastric emptying are associated with feelings of fullness and satiation (53, 54). In this trial, erythritol induced an increase in fullness and a decrease in prospective food consumption. The findings are most likely related to the observed release of GI satiation hormones and the retardation of gastric emptying. In contrast to erythritol, D-allulose did not affect appetite-related sensations despite the marked increase in GI satiation hormones. As discussed above, changes in gastric emptying play an important role in the regulation of hunger and satiety feelings. The missing effect on gastric emptying observed in response to D-allulose is in line with this observation.

The mild and short-lasting symptoms of the present study for D-allulose are in line with a previous GI tolerance study (30). There was a slight increase in symptoms after the erythritol-containing solutions compared with our most recent study (29). However, participants familiarized to erythritol intake show a higher GI tolerance (55). The participants in this trial were not used to these substances, and the test solutions were rapidly applied (over 2 min) immediately into the stomach, which probably causes the greatest stress for the GI tract.

Some limitations of our study require consideration: first, we studied acute effects of single-bolus doses of D-allulose and erythritol with and without lactisole applied in a liquid solution to participants with a BMI between 19.0 and 24.9 who were not used to these substances. Differential effects of long-term exposure on the secretion of GI satiation hormones and gastric emptying rates need to be investigated, as adaptive processes cannot be ruled out. Second, we measured total GLP-1, which may imply less sensitivity toward detecting a small size effect for the gut sweet taste receptor inhibition than active GLP-1. Third, the substances used in this trial may behave differently when included in a food matrix with other nutrients rather than administered in isolation. Moreover, effects on subsequent food intake were not measured. Fourth, appetite-related sensations could have been affected by the presence of the feeding tube, although in the present study, it was used for only a short period of time and immediately removed after the administration of the test solutions.

In conclusion, D-allulose and erythritol stimulate the secretion of GI satiation hormones in humans. Lactisole had no effect on CCK, GLP-1, and PYY release, indicating that D-allulose– and erythritol-induced GI satiation hormone release is not mediated via the gut sweet taste receptor (T1R2/T1R3). The mechanism remains to be determined.

## Data Availability

Data described in the manuscript and code book will be made publicly and freely available at https://github.com/labgas/proj_erythritol_1.

## Notes

Supported by the Swiss National Science Foundation and the Research Foundation Flanders (grant 320030E_189329) to ACM-G, BKW, and LVO.

Author disclosures: The authors report no conflicts of interest.

BKW and ACM contributed equally to this work.

Abbreviations used: CCK, cholecystokinin; EEC, enteroendocrine cell; GI, gastrointestinal; GLP-1, glucagon-like peptide 1; ppm, parts per million; PYY, peptide tyrosine tyrosine; SGLT-1, sodium-glucose transporter 1; T2DM, type 2 diabetes mellitus; VAS, visual analog scale.
